# Inteins as Drug Targets and Therapeutic Tools

**DOI:** 10.3389/fmolb.2022.821146

**Published:** 2022-02-08

**Authors:** Anil Mathew Tharappel, Zhong Li, Hongmin Li

**Affiliations:** ^1^ Department of Pharmacology and Toxicology, College of Pharmacy, The University of Arizona, Tucson, AZ, United States; ^2^ BIO5 Institute, The University of Arizona, Tucson, AZ, United States

**Keywords:** intein, inhibitor, drug target, therapeutic tool, anti-microbial

## Abstract

Multidrug-resistant pathogens are of significant concern in recent years. Hence new antifungal and anti-bacterial drug targets are urgently needed before the situation goes beyond control. Inteins are polypeptides that self-splice from exteins without the need for cofactors or external energy, resulting in joining of extein fragments. Inteins are present in many organisms, including human pathogens such as *Mycobacterium tuberculosis*, *Cryptococcus neoformans*, *C. gattii*, and *Aspergillus fumigatus*. Because intein elements are not present in human genes, they are attractive drug targets to develop antifungals and antibiotics. Thus far, a few inhibitors of intein splicing have been reported. Metal-ions such as Zn^2+^ and Cu^2+^, and platinum-containing compound cisplatin inhibit intein splicing in *M. tuberculosis* and *C. neoformans* by binding to the active site cysteines. A small-molecule inhibitor 6G-318S and its derivative 6G-319S are found to inhibit intein splicing in *C. neoformans* and *C. gattii* with a MIC in nanomolar concentrations. Inteins have also been used in many other applications. Intein can be used in activating a protein inside a cell using small molecules. Moreover, split intein can be used to deliver large genes in experimental gene therapy and to kill selected species in a mixed population of microbes by taking advantage of the toxin-antitoxin system. Furthermore, split inteins are used in synthesizing cyclic peptides and in developing cell culture model to study infectious viruses including SARS-CoV-2 in the biosafety level (BSL) 2 facility. This mini-review discusses the recent research developments of inteins in drug discovery and therapeutic research.

**Graphical Abstract:**
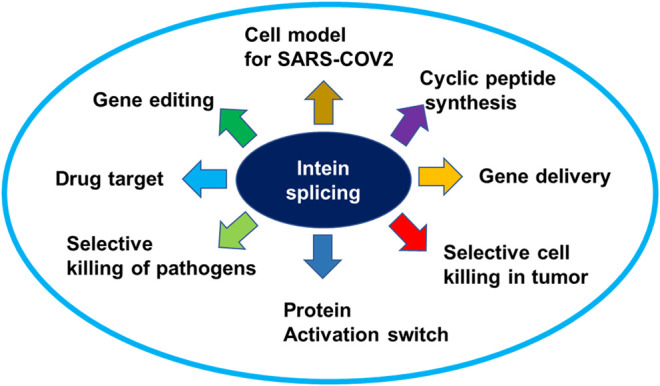


## Introduction

Prolonged use of drugs can lead to drug-resistant strains of pathogens and is a major challenge in treating the diseases. Drug resistance have been reported in *Mycobacterium tuberculosis, Cryptococcosis neoformans* and *C. gattii* that are causative agents of tuberculosis (TB) and cryptococcosis, respectively. In fact, antimicrobial resistance has been determined as one of the top 10 global public health threats by the World Health Organization (WHO) (WHO, 2021a). Although TB has been declining over the years, around 10 million people caught the disease with 1.5 million deaths reported in 2020 (Furin et al., 2019; WHO, 2021b). Since the start of antibiotic treatment in 1943, some strains of bacteria developed resistance to first-line anti-TB drugs, isoniazid and rifampin. The majority of the multi-drug resistant strains of *M. tuberculosis* are of Beijing lineage (Parwati et al., 2010; Stoffels et al., 2013). Similarly, there are reports of drug resistance to frontline antifungal drugs such as amphotericin B, fluconazole and 5-fluorocytosine (Bandara et al., 2020; Billmyre et al., 2020; Carolus et al., 2020). Although many antifungals are in the pipeline (Gintjee et al., 2020), the discovery of new targets will help to develop alternative strategies to combat the diseases (Su et al., 2018).

Inteins are small mobile elements within a host protein. Inteins can self-splice without external energy or cofactors and ligate the host protein fragments to generate active proteins (Pinto et al., 2020). Intein was first reported from the protein vacuolar membrane H^+^-translocating adenosine triphosphatase (*VMA1* or *TFP1*) gene of *Saccharomyces cerevisiae* (Hirata et al., 1990). Inteins can be classified by its structural components or by splicing mechanisms. There are mainly three types of inteins based on the structural components prior to splicing (Lin et al., 2013). The max-intein has a homing endonuclease (HE) domain (Singh et al., 2009) which can hydrolyze genomic DNA within the cells (Chevalier and Stoddard, 2001), whereas mini-inteins do not have HE (Volkmann and Mootz, 2013). The third type is split inteins which have two fragments; the N terminal fragment joins to the N-terminal extein, whereas the C-terminal fragment links to the C terminal extein (Wu et al., 1998). Once the two fragments of split intein assemble, the split intein performs regular splicing activity (Beyer et al., 2020).

The splicing of intein is a rapid reaction. Hence rarely the precursor protein is observed in its native form. The active site is formed by folding of intein within the precursor, resulting in splice junctions (Tori et al., 2010). The intein and the first C-extein amino acid (aa) together act as a single turnover enzyme for splicing. Based on the splicing mechanisms, there are three classes of inteins (Tori et al., 2010; Nanda et al., 2020). Most inteins fall under class I inteins that most commonly have Ser, Thr, Cys, or Asn as essential residues that act as nucleophiles during splicing. The class I intein splicing consists of four coordinated nucleophilic displacement reactions. The reactions are: 1) amide (thio)ester and rearrangement, 2) transesterification and branch formation, 3) Asn cyclization and branch resolution, 4) acyl rearrangement or succinimide hydrolysis (Mills et al., 2014). Class II and III inteins have an Ala at their N-termini (Basturea, 2021). These two class inteins also form branched intermediate present in class I intein, but do so by different pathways as the N-terminal Ala cannot form linear thioester intermediates. The class II inteins skip the first step of class I intein splicing in which the intein N-terminus Cys/Ser residue directly attacks the N-terminal splice site amide bond to form a Block G branched intermediate (Mills et al., 2014). The class III inteins form a specific Block F branched intermediate with Cys at block F as the branch point before arrive at the Block G branched intermediate (Tori and Perler, 2011a). The active site residues and positions within the intein vary in each classes (Tori and Perler, 2011b).

The presence or absence of intein is species dependent. A total of 2,709 intein-containing genomes were found from the NCBI database, out of which 56% were found in eukaryotes, 19.8% in archaea, 6.64% in eubacteria and 17.4% in viruses (Nanda et al., 2020). Most of the inteins are located at conserved sites of housekeeping proteins with important functions, such as aminoacyl tRNA synthetases, DNA and RNA polymerases, recombinases, helicases topoisomerases, and spliceosomal components (Novikova et al., 2014; Fernandes et al., 2016).

### Inteins in Human Pathogens

Many human pathogens have intein elements in their genes (Table 1A). Among infectious diseases, TB is one of the significant causes of human death worldwide. The causative agent *M. tuberculosis* has one intein in each of the three *M. tuberculosis* proteins: replicative helicase (DnaB), recombinase (RecA), and an iron-sulfur cluster assembly (SufB) (Topilina et al., 2015). In contrast, closely related nonpathogenic *M. smagmatis* has only two inteins Dnabi1 and Dnabi2, in DnaB (Kelley et al., 2018). The DnaB helicase unwinds DNA from 5′ to 3′ direction at the replication fork, which is critical in replication initiation (LeBowitz and McMacken, 1986; Bailey et al., 2007). *M. laprae*, the causative agent of leprosy, has one intein in each of its DnaB, RecA, SufB and GyrA proteins (Table 1A). Although the RecA protein of *M. leprae* is structurally analogous to that of *M. tuberculosis,* it functions differently from its counterpart in *M. tuberculosis* (Patil et al., 2011). RecA deletion studies in *E. coli* (Kurnit, 1989) and *M. smegmatis* (Papavinasasundaram et al., 1998) indicate that RecA is not necessary for survival. In contrast, both DnaB and SufB (Huet et al., 2006) are essential for *M. tuberculosis*.

**TABLE1:** Inteins in human pathogens and intein splicing inhibitors.

A	Intein-containing human pathogens of fungal and bacterial origin					
Disease	Causative agent	Details of intein				References
		Name	Aa	E/T	HE	
Tuberculosis	*M. tuberculosis*	DnaB	416	E	P	Paulus (2003)
		RecA	440	E	P	Daugelat and Jacobs (1999), Saves et al. (2002), Paulus. (2003), Zhang et al. (2010), Chan et al. (2016)
		SufB	359	T	P	Saves et al. (2002), Huet et al. (2006), Topilina et al. (2015)
Leprosy	*M. leprae*	DnaB	145	E	A	Eiglmeier et al. (1993), Lennon et al. (2021)
		RecA	365	T	P	Davis et al. (1994), Singh et al. (2009)
		SufB (Pps1)	386	T	P	Smith et al. (1997), Saves et al. (2002)
		GyrA	420	E	P	Fsihi et al. (1996)
Pulmonary infection	*M. xenopi*	GyrA	198	E	A	Telenti et al. (1997), Klabunde et al. (1998), Roque, (2020)
Q fever	*Coxiella burnetii*	DnaB	146	T	A	Seshadri et al. (2003), Raghavan et al. (2008), Neupane and Kaswan (2021)
Cryptococcosis	*C. Neoformans-*JEC21	Prp8	172	T	A	Liu and Yang. (2004), Butler et al. (2006)
	*C. neoformans grubii*	Prp8	171	E	A	Butler et al. (2006)
	*C. gatti*	Prp8	170	T	A	Butler et al. (2006)
Fungemia	*C. laurentii*	Prp8	522	T	P	Butler and Poulter. (2005), Salazar-Leal et al. (2019)
Aspergillosis	*A. fumigatus*	Prp8	819	T	P	Liu and Yang (2004)
Affects CGD patients	*A. nidulans*	Prp8	605	E	P	Butler et al. (2006), Bastos et al. (2020)
Histoplasmosis	*Histoplasma capsulatum (Ajellomyces capsulatus)*	Prp8	534	E	P	Grainger (2005)
Paracoccidioido-mycosis	Paracoccidioides brasiliensis	Prp8	573	T	P	Theodoro et al. (2011)
Blstomycosis	*Blastomyces dermatitidis*	Prp8	526	E	P	Theodoro et al. (2011)
Adiaspiromycosis	*Emmonsia parva*	Prp8	526	E	P	Theodoro et al. (2011)
Emergomycosis	Emergomyces pasteurianus	Prp8	549	T	P	Chaturvedi and de Hoog. (2020), Garcia Garces et al. (2020), Samaddar and Sharma (2021)
	Es. africanus	Prp8	577	T	P	Dukik et al. (2017), Garcia Garces et al. (2020)
	Es. orientalis	Prp8	582	T	P	Dukik et al. (2017), Garcia Garces et al. (2020)

B	Inhibitors of intein/hedgehog splicing and targets	
Compound name	Target species (Intein/Hedgehog)	References
Cisplatin	*M. tuberculosis* (RecA)	Zhang et al. (2011), Li et al. (2019)
	*C. neoformans* (Prp8)	
	*C. gattii* (Prp8)	
6G-318S	*C. neoformans* (Prp8)	Li et al. (2021)
	*C. gattii* (Prp8)	
6G-319S	*C. neoformans* (Prp8)	Li et al. (2021)
ST044643	*Drosophila* (Hedgehog)	Owen et al. (2015)
Zn^2+^	*M. smegmatis* (DnaB)	Woods et al. (2020), Butler and Poulter. (2005); Xie et al. (2015)
	*M. lapre* (DnaB)	
	*Drosophila* (Hedgehog)	
Cu^2+^	*Drosophila* (Hedgehog)	Xie et al. (2015)
	*M. smegmatis* (RecA)	Zhang et al. (2010)
H_2_O_2_	*M. tuberculosis* (RecA)	Kelley et al. (2018)

Note: aa: amino acid; HE: Homing endonuclease domain; P: Present; A: Absent; CGD: Chronic granulomatous disease; T: Theoretical; E: Experimental.

*Coxiella burnetii* is considered a re-emerging zoonosis in many countries. *C. burnetii* naturally infects livestock animals, such as goats, sheep, and cattle (Abdel-Moein and Zaher, 2021). It causes Q fever in human. In 2019, 178 acute and 34 chronic Q fever cases were reported in USA (CDC, 2021). *C. burnetii* has a DnaB intein with an approximate size of 16 kDa (Raghavan et al., 2008).

In the fungal kingdom, the pre mRNA processing factor 8 (Prp8) intein is the most widespread (Theodoro et al., 2013). Active Prp8 is critical for eukaryotic spliceosome responsible for pre-mRNA splicing (Grainger, 2005). The human version of Prp8 is also known by other names such as PRPF8, PRPC8, p220, and 220K in literature (Grainger, 2005). Some fungal pathogens such as *C. neoformans* and *C. gattii* have mini inteins without the HE domains in their Prp8 proteins, whereas *A. fumigatus* and *Histoplasma capsulatum* have the HE domains in their inteins (Table 1A).

Emergomycosis is an emerging disease caused by a novel dimorphic fungus Emergomyces species in immunocompromised individuals (Samaddar and Sharma, 2021). Due to taxonomic similarity, Emergomyces was under genus Emmonsia. As per recent classification, Emergomyces genera include *E. pasteurianus, E. africanus, E. canadensis, E. orientalis*, and *E. europaeus* (Jiang et al., 2018; Schwartz et al., 2019), many of which have inteins in their Prp8 proteins (Garcia Garces et al., 2020) (Table 1A).

### Inteins as Drug Targets

Many human pathogens contain inteins in some of their proteins that are crucial for survival. Therefore, inhibition of intein splicing will be an attractive strategy, especially when drug resistance is reported for frontline therapeutic agents. The absence of inteins in human proteins is an added advantage in targeting pathogens. Below is a summary of currently known intein inhibitors (Table 1B).

#### Metal Ion and Metal-Compounds as Intein Inhibitors

**FIGURE 1:**
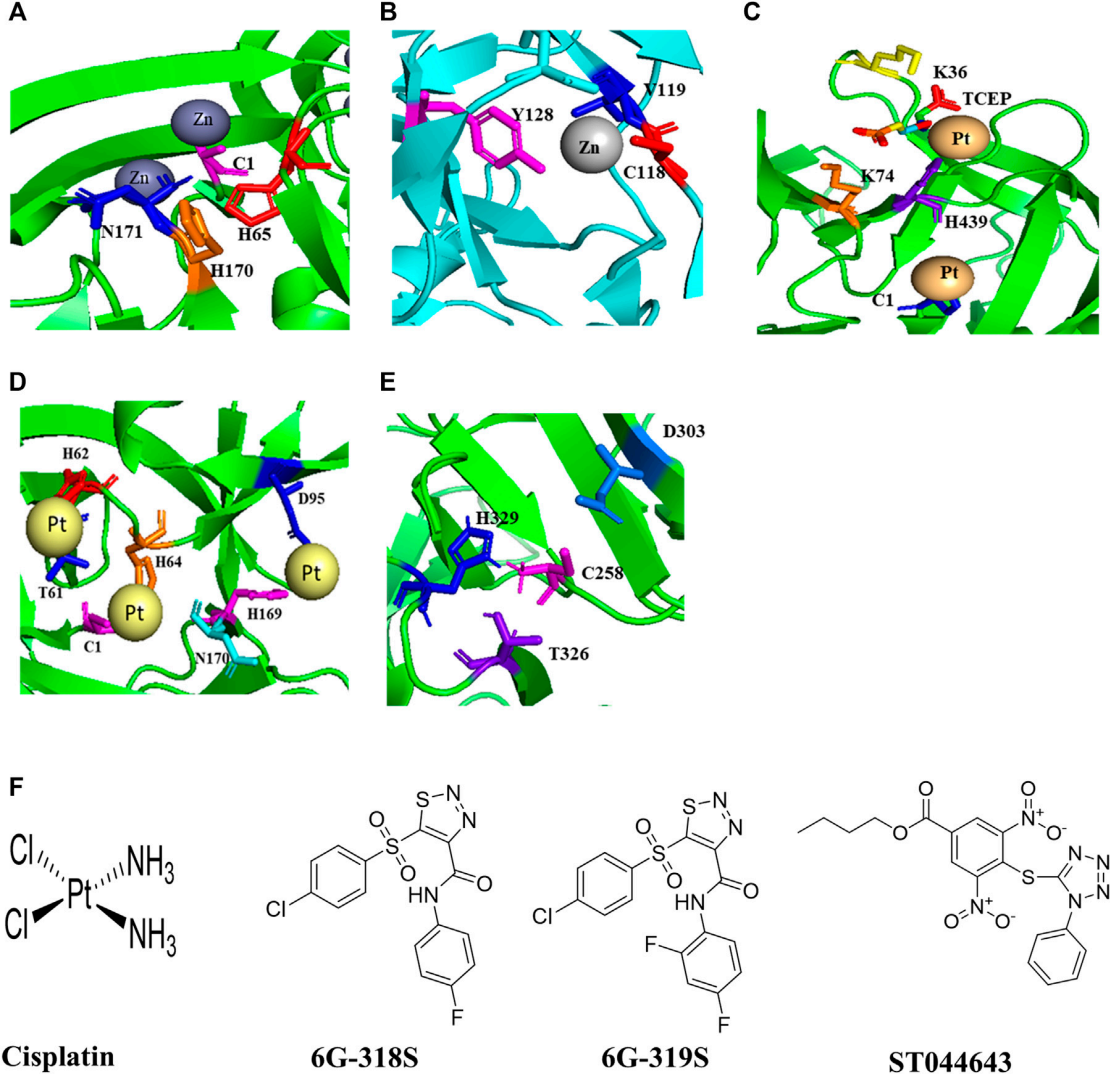
Structures of inteins, intein-ligand complexes, and small molecule inhibitors of intein splicing and hedgehog cholesterolysis. **(A)** The Prp8 intein of *C. neoformans* and Zn^2+^ (Green et al., 2019). **(B)** The DnaBi1 intein of *M. smegmatis* and Zn^2+^ (Woods et al., 2020). **(C)**, The RecA intein of *M. tuberculosis* in complex with platinum and TCEP (Chan et al., 2016). **(D)** The Prp8 intein of *C. gattii* and cisplatin (Li et al., 2019). **(E)** The C-terminal 17 kDa fragment of drosophila hedgehog showing catalytic residues in cholesterolysis (Hall et al., 1997). **(F)** Structures of small molecules that inhibit intein splicing and hedgehog cholesterolysis.

Biologically relevant metal ions Cu^2+^ and Zn^2+^ at 0.5 mM and 2 mM respectively could inhibit splicing of the *C. neoformans* Prp8 intein in an *in vitro* Prp8 intein splicing assay, whereas Mg^2+^ at 0.5 mM and 2 mM did not (Green et al., 2019). ZnSO_4_ binds with the Prp8 intein of *C. neoformans* with a binding affinity K_D_ of 1 ± 0.8 nM in an isothermal titration calorimetry assay. The crystal structure of the Prp8 intein in complex with Zn^2+^ shows that C1, H65, H170, N171 are involved in Zn^2+^ binding (Figure 1A). The mechanism of action of Zn^2+^ and Cu^2+^ seems different. Cu^2+^ likely stimulates reversible modifications on catalytically active cysteine, whereas Zn^2+^ binds at the terminal asparagine and the critical cysteine, resulting in inhibition of splicing (Green et al., 2019).

Zn^2+^ also reversibly inhibits the splicing of DnaBi1 of *M. smegmatis.* In a splicing assay where the intein is placed between MBP and GFP (MIG), complete inhibition of DnaBi1splicing was observed at 10 µM of Zn^2+^. The metal-chelator EDTA could reverse the splicing (Woods et al., 2020)*.* Zn^2+^ also inhibits splicing of the DnaB intein of *M. laprae* (Woods et al., 2020) which is homologous to the *M. smegmatis* DnaBi1 (Kelley et al., 2018). Additionally, Zn^2+^ could inhibit splicing *in vivo* in *M. smegmatis.* DnaBi1 and the *M. leprae* DnaB intein share 68.0% sequence identity. DnaBi2 and the *M. tuberculosis* DnaB intein have 61.0% amino acid identity (Kelley et al., 2018). A 1.95 Å crystal structure of *M. smegmatis* DnaBi1 indicates that the sulfur on C118, peptide backbone atoms N and O of V119, and the hydroxyl group of Y128 contributed to the coordination of the Zn^2+^ ions (Figure 1B) (Woods et al., 2020)*.* The DnaBi1 of *M. smegmatis* responds to oxidative stress, whereas DnaBi2 is not (Kelley et al., 2018). Cd^2+^, Ni^2+^ and Co^2+^ have also shown inhibitory activity, although weaker than Zn^2+^, in splicing of the RecA intein from *M. tuberculosis* and of naturally split DnaE intein from *Synechocystis* sp., whereas there is no inhibition by Mg^2+^ and Ca^2+^ (Mills and Paulus, 2001; Panda et al., 2021).

Cisplatin, a platinum-containing anticancer drug, inhibits the RecA intein splicing with an IC_50_ of 2 µM in an *in vitro* splicing assay. The minimum inhibitory concentration (MIC) of cisplatin against *M. tuberculosis* was 40 µM (Zhang et al., 2011). Platinum-based compounds Pttfbz and Zeise’s salt have similar IC_50_ values (1.97, and 1.18 μm, respectively) as that of cisplatin (1.67 µM) in split GFP based assay, which employs a minimized *M. tuberculosis* RecA intein (Chan et al., 2016). Cisplatin and Pttfbz bind to intein in the presence of TCEP, whereas Zeise’s salt binding is independent of TCEP (Chan et al., 2016). The interaction of TCEP with cisplatin is also reported previously (Boal and Rosenzweig, 2009; Chen et al., 2013). The crystal structure of an HE-less RecA in complex with cisplatin and TCEP was solved at 1.50 Å (Figure 1C) (Chan et al., 2016). The cisplatin may modify one of the cysteine residues in the active site of the RecA intein (Zhang et al., 2011).

Cisplatin also inhibits the *C. neoformans* Prp8 intein splicing with IC_50_ of 2.5 µM in an *in vitro* splicing assay based on split *Renilla* luciferase; and the MIC_90_ was 4.5–20 μg/ml in various strains of *C. neoformans* and *C. gattii* (Li et al., 2019). It was reported that cisplatin treatment at 8 mg/kg once daily for 4 days led to two log-order reductions of *C. neoformans* in the lungs of BALB/c mice that were challenged with H99 at 1 × 10^7^ CFU/mouse (Li et al., 2019). The crystal structure of the *C. gatti* Prp8 intein in complex with cisplatin indicates that the residues C1, T61, H62, H64, H169, N170, D95 are involved in the complex formation (Figure 1D) (Li et al., 2019). Mutagenesis of active site residues C1 and H169 abolished the binding of cisplatin to the Prp8 intein.

#### Non-Metal Small Molecule Inhibitors

In addition to metal-containing inhibitors, reactive nitrogen species compounds (DEA NONOate at 1.2 mM and 12 mM) and Angeli’s salt at 2 mM and 20 mM) were found to inhibit splicing of the *C. neoformans* Prp8 intein in an *in vitro* Prp8 intein splicing assay, whereas H_2_O_2_ (.8 and 8 mM) did not (Green et al., 2019). In contrast, H_2_O_2_ at 8 mM concentration inhibited the splicing of *M. smegmatis* DnaBi1 in the MIG splicing assay *in vitro* (Kelley et al., 2018). Moreover, H_2_O_2_ at 5 mM abolished splicing of the DnaB precursor *in vivo* in *S. smegmatis* in a western blot assay using an anti-DnaB extein antibody (Kelley et al., 2018).

From a small-scale screening of small molecules using split luciferase and split GFP-based *C. neoformans* Prp8 intein splicing assays, a compound 6G-318 was found as an inhibitor of intein splicing with an IC_50_ of 5.8 µM and 11.2 µM, respectively (Li et al., 2021) (Figure 1F). When tested in *C. neoformans*, the compound displayed MIC values of 0.62–1 μg/ml. Out of the four commercially available derivatives, 6G-319S, 12G-305S, 6G-313S, and 12G-315S, only the fluoride derivative 6G-319 was active against *C. neoformans* with MIC of 1.3 μg/ml (Figure 1F). 6G-318S has shown a synergistic effect with amphotericin B. The cytotoxicity CC_50_ was determined for the adenocarcinomic human alveolar basal epithelial cells (A549) cells. The ratio of CC_50_ to MIC for 6G-318S and 6G-319S were 22.4 and 34.5, respectively. The inhibition of intein splicing was further demonstrated by an *in vitro* MIG splicing assay and *in vivo* in *C. neoformans*. The binding of 6G-318S to the Prp8 intein was demonstrated by thermal shift assay, mass-spectrometry, and surface plasmon resonance (SPR) assays. The dissociation constant K_D_ of 6G-318S was .36 µM for wild type Prp8 intein in the SPR assay (Li et al., 2021).

### Intein as a Tool in Therapeutics and Drug Discovery

#### Split Inteins

Split-inteins express as two separate polypeptides at the ends of two host proteins and catalyze their trans-splicing, resulting in the formation of a single larger polypeptide (Li, 2015). Translation of two genes happens separately. After translation, two intein parts ligate their flanking protein parts to each other, resulting in mature protein (Caspi et al., 2003). Naturally, split-inteins are found only in the DNA polymerase III alpha subunit (polC or dnaE gene) of some cyanobacteria (Caspi et al., 2003). The DnaE intein family of cyanobacteria are the largest known class of split inteins (Caspi et al., 2003).

#### Split Inteins in Gene Therapy, Gene Delivery, and Gene Editing

**FIGURE 2:**
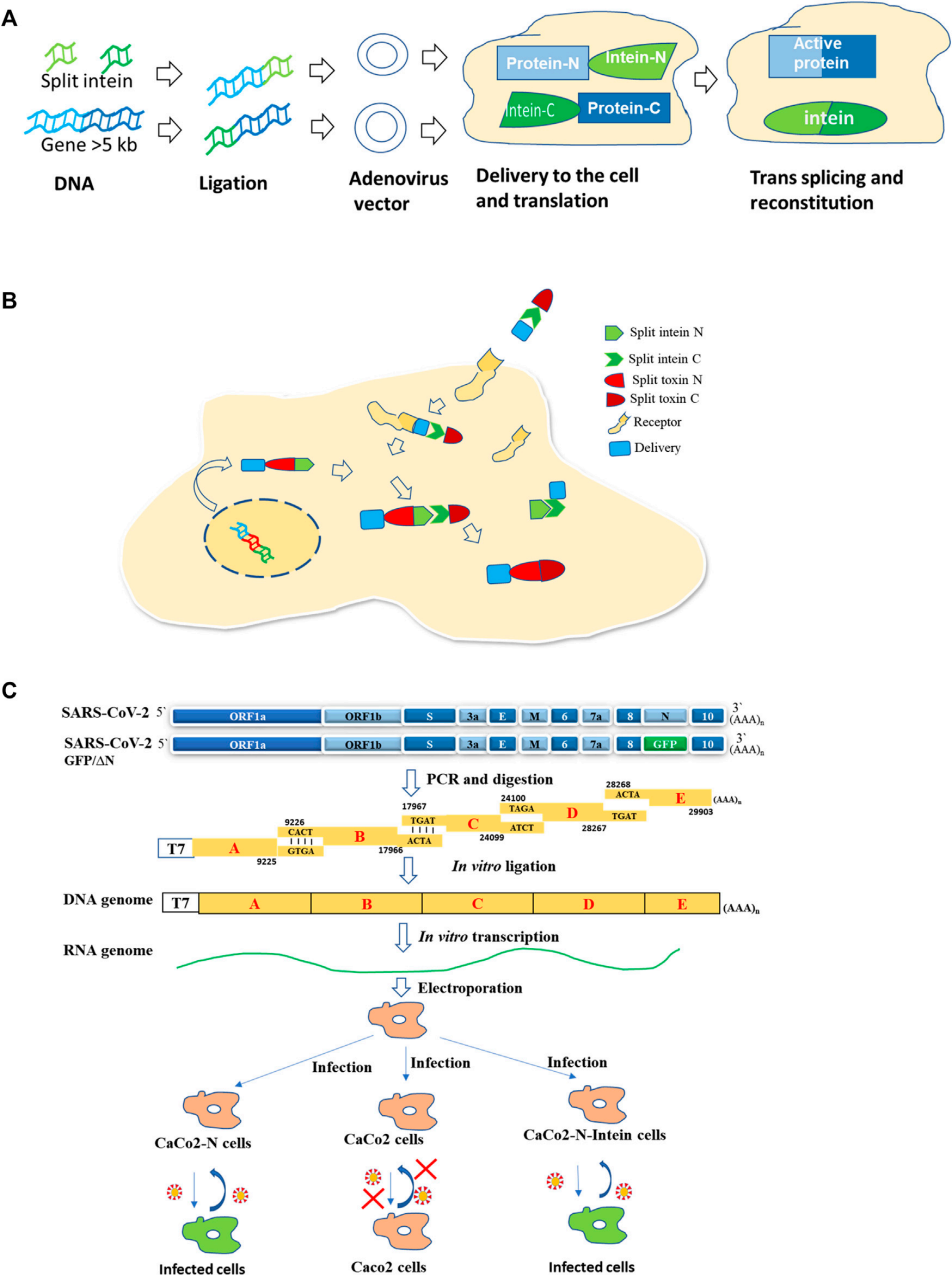
Use of split intein in therapy and pathogenesis studies. **(A)** Large gene delivery in gene therapy (Tornabene et al., 2019; Lim et al., 2020). **(B)** Intein mediated toxin reconstitution inside the cells for selective cell killing in tumor therapy (Purde et al., 2020). **(C)** Development of a BSL2 cell culture model for SARS-COV-2 in CaCo2 cells (Ju et al., 2021).

Inteins are being used for various biotechnological applications (Shah and Muir, 2014). One such application is gene therapy which is gaining importance. Gene therapy is promising for various diseases. Adeno-associated viral (AAV) vectors mediated retinal gene therapy promises to treat inherited and non-inherited eye diseases. The delivery of genes that are large (>5 kb) is a challenge. AAV vectors with fragments of target proteins flanked by short split inteins result in protein trans-splicing, leading to reconstitution of full-length protein in the retina of mice and pigs, and in human retinal organoids (Tornabene et al., 2019) (Figure 2A).

A similar methodology was used for expressing large genes in several studies. Using CRISPR/Cas9, one can target any genes, but the size of Cas9 is a limitation. The coding sequence for Cas9 is divided into two parts on a dual vector having split intein fragments, which will get reconstituted post-translationally without affecting its endonuclease activity (Truong et al., 2015). Furthermore, the methodology was used for CRISPR-based editors to treat amyotrophic lateral sclerosis (ALS), which is due to mutations in the superoxide dismutase 1 (SOD1) gene in a G93A-SOD1 mouse model of ALS (Lim et al., 2020). Dual AAV particles encoding split intein engineered to trans-splice and substitute a mutant SOD1 gene resulted in prolonged survival, slow disease progression, reduced muscle atrophy, improved neuromuscular function, and 40% fewer SOD1 immunoreactive inclusions as compared to control mice (Lim et al., 2020). Similar treatments were made using split inteins in dual AVV1 vectors to replace the mutated dystrophin gene in Duchenne muscular dystrophy (DMD) (Li et al., 2008). DMD is a common and lethal childhood muscle disorder, affecting 1 in every 3,500 male births (Koenig et al., 1987). After confirming the Becker-form dystrophin protein that has X chromosome-linked mutation in cell culture *in vitro*, AAV1 vectors were transferred into the muscle of Duchenne muscular dystrophy mouse model, resulting in therapeutic gene expression and benefits (Li et al., 2008).

The split inteins were also used for gene editing. Point mutations are seen in pathogenic human genetic variants. Adenine base editors (ABEs) catalyzes target A·T base pairs to G·C; and cytosine base editors (CBEs) converts target C·G base pairs to T·A. Although many studies have been done with base editors (Porto et al., 2020), the size of base editors is a limitation. To solve the issue, both CBE and ABE can be divided and fused to split intein fragments in an AAV vector system to form full base editors after trans splicing reconstitutes. This type of editing was done in somatic tissues liver, heart, muscle, retina, and brain that have relevance in therapeutic (Levy et al., 2020). Moreover, dual-AAV split-intein base editors have been used to treat Niemann-Pick disease type C that affects the central nervous system in a mouse model (Levy et al., 2020). In another study, the clotting factor VIII fused with split inteins was delivered into HEK293 and Cos-7 cells as a proof of concept. Lack of clotting factor 8 is seen in a hereditary bleeding disorder called Hemophilia A (Zhu et al., 2010). Split inteins were also used for treating experimental retinal Stargardt macular dystrophy (SMD), an inherited macular dystrophy in humans. In SMD, the ATP binding cassette subfamily A member 4 (ABCA4) is mutated. ABCA4 is involved in the clearance of photoisomerized all-trans-retinal from the photoreceptor disk lumen. Mutated ABCA4 results in a buildup of lipofuscin pigments in the retinal pigment epithelial cells, causing vision loss in patients with Stargardt disease (Tornabene et al., 2021). In order to repair the ABCA4 mutation, the ABCA4 gene is split into two; and each half is linked to split inteins which are separated, loaded onto AVV vectors, and delivered to one-month-old Abca4^−/−^ mice subretinally. As expected, lipofuscin was observed to accumulate in the retinal pigmented epithelium of the vehicle-treated Abca4^−/−^ mice. In contrast, there was a significant reduction in lipofuscin, in addition to improvement in other phenotypes for the mice treated with ABCA4-split intein after 3 months (Tornabene et al., 2021). Similarly, studies with the retina of pig and human retinal organoids also showed improvement for the ABCA4-split-intein group compared with the vehicle controls (Tornabene et al., 2021).

One of the issues is that non-mammalian origin components of AAV vectors could elicit immune and toxic responses in target cells or raise regulatory concerns for clinical use. To overcome this issue, a degron can be included in the trans-splicing system. The specific signals which turn the protein susceptible to ubiquitin-mediated proteasomal degradation are called degrons (Varshavsky, 1991). Inclusion of degron *E. coli* dihydrofolate reductase (ecDHFR) in the N-intein results in selective degradation of excised inteins from the AVV vector that is used for delivery of the ABCA4 gene for retinal therapy (Tornabene et al., 2021). The degradation ability of ecDHFR is inhibited by a small stabilizing ligand, trimethoprim.

#### Split Inteins in Anti-Tumor Therapy

Split intein was used to reconstitute a toxin inside selected cells enabling selective cell killing in mixed populations and tumor xenografts (Purde et al., 2020). The Diphtheria toxin catalytic domain (DTA) was split into two, with each fused to split intein from *N. punctiforme*. Split-toxinN encoding DNA is delivered by transfection/viral transduction, while recombinant split toxin C is delivered *via* a specific receptor by the anthrax toxin translocation system. The active toxin will be formed intracellularly by intein-mediated trans-splicing of two split-toxin parts (Purde et al., 2020) (Figure 2B). The delivery of split toxin *via* cell surface-specific receptors will help in the killing of specific cell populations. An attempt of HER2 receptor-mediated delivery was also made to deliver a split *Pseudomonas aeruginosa* exotoxin, although it was reconstituted first *in vitro* outside the cell (Wang et al., 2019).

#### Split Inteins to Study Microbial Pathogenesis

One of the drawbacks of broad-spectrum antibiotics is dysbiosis and concomitant health sequelae. Human gut has around 100 trillion microbes from over 1,000 species (Zhang and Chen, 2019). Targeted killing of harmful bacteria without harming beneficial ones can reduce dysbiosis and drug resistance. With the help of split intein, López-Igual et al. (2019) developed a toxin against *V. cholerae* using a toxin-antitoxin (TA) system. Various TA systems are present in most of prokaryotes. Toxins are proteins that reduce metabolism, whereas antitoxins are either RNA or proteins that counteract toxins (Song and Wood, 2020). TA is involved in the stabilization and fitness of mobile DNA, genome stabilization, and phage protection. The *M. tuberculosis* has 85 TA modules, whereas nonpathogenic *M. smegmatis* has only 5 TA modules (Yu et al., 2020). The TA toxins are different from endotoxin and exotoxins. The TA toxins function only inside the cells that produce TA and are not secreted outside the cells (Singh et al., 2021). The TA system is grouped into Type I to Type VI based on the mode of action of antitoxin (Harms et al., 2018). Over expression of toxins is bactericidal. The CcdA/CcdB Type II TA system is one example of the bacterial TAs. In order to design a toxin–intein antimicrobial which is *V. cholerae* specific, the type II toxin gyrase poison CcdB is split and fused to the split intein DnaE of *N. punctiforme.* Antibiotic-resistant *V. cholerae* bacteria receiving the toxin-intein containing plasmid are completely killed at its hosts such as zebrafish and crustacean *Artemia salina* (López-Igual et al., 2019).

The protein trans-splicing is also utilized to generate a novel cell culture model for SARS-COV2, which consists of viral RNA without N capsid and a producer cell line expressing viral N protein. In this system, two fragments of N are linked to split intein fragments. Ligation takes place to produce full-length N protein (Ju et al., 2021) (Figure 2C). This model will help in the study of viral pathogenesis as well as in screening antivirals in a BSL2 facility.

#### Inteins in Conditional Drug Delivery and Peptide Synthesis

Engineered full-length inteins can also be employed to activate protein of interest using small molecules such as rapamycin (Mootz and Muir, 2002) and 4-hydroxytamoxifen (4-HT). 4-HT is an agonist of estrogen receptor (ER) (Sasson and Notides, 1988) and is cell-permeable. By inserting a natural ligand-binding domain (LBD) into a mini-intein can destroy splicing activity (Buskirk et al., 2004). A modified RecA can get spliced in the presence of 4-HT. The HE of the RecA is replaced with LBD of ER, which can be placed in any protein to facilitate ligand-dependent splicing using 4-HT. The splicing was 50–90% in the presence of 4-HT and <5% in the absence of 4-HT (Peck et al., 2011). These inteins may help in the modulation of protein activities post-translationally in living systems such as mammalian cells and help *in vivo* activation of intein-fused therapeutic proteins.

Split-intein circular ligation of peptides and proteins (SICLOPPS) is used to develop macrocyclic peptides inside cells and to phenotypically screen cells for them. The split inteins are fused to C and N termini of the target peptide. Upon trans splicing, circular peptide is formed (Scott et al., 1999). Circular peptides are more resistant to protease activity and may be suitable for oral administration as a drug (Tavassoli, 2017; Muttenthaler et al., 2021).

## Discussion

Drug resistant strains of pathogens are reported in many disease outbreaks. In the year of 2018, there were about half a million new cases of rifampicin-resistant TB globally, the majority of which have multi-drug resistant TB (MDR-TB), a TB form resistant to two or more anti-TB drugs. TB was one of the major infectious killer worldwide prior to the COVID-19 pandemic (WHO, 2021b).

The distribution of intein is species-specific. It is still not clear why intein persists for millions of years of evolution (Novikova et al., 2016; Nanda et al., 2020). Although the exact functions of inteins remain elusive, splicing of intein from exteins is essential for activity of the intein-harboring protein. Many bacterial and fungal pathogens harbor inteins in their essential proteins. Since DnaB is an essential protein, inhibition of intein splicing can inhibit the growth of *M. tuberculosis*. Nonpathogenic *M. smegmatis* is frequently used as a model to study *M. tuberculosis*. Presence of the DnaB intein in *M. smegmatis* will facilitate the research on intein as it can be done in a BSL1 facility.

Another group of disease-causing organisms that have inteins are fungal pathogens. Although immunocompromised individuals are major concern for fungal infection, immunocompetent individuals are also susceptible to fungal species such as *C. gattii* (Andreou et al., 2019). Thus, it is essential to develop more antifungals with novel mechanisms of action such as *via* inhibition of intein splicing. Significant progress has been made recently in identification of inhibitors of intein splicing. Non-metal small molecule inhibitors started to emerge, showing inhibition of intein splicing both *in vitro* and *in vivo* (Li et al., 2021). Further studies will be required to identify more potent candidate intein splicing inhibitors and/or to optimize existing inhibitors to achieve clinical significance to combat infections of intein-containing microbes. It is noted that intein splicing inhibitors may be limited to intein-containing microbes, and may not be suitable for non-intein containing fungal pathogens such as *Candida albicans* (Yapar, 2014; Fernandes et al., 2016).

No intein has been reported in humans. However, an autoprocessing mechanism is found for cholesterolysis of the human Hedgehog (Hh) protein, which mimics intein splicing. The Hh protein is synthesized as a 45 kDa precursor, undergoes auto-processing to yield a 25 kDa C-terminal fragment and a 20 kDa N-terminal fragment with cholesterol moiety covalently attached to it. Therefore, it is necessary to check the off-target activity of the intein inhibitors (Perler, 1998) with human Hh protein. The crystal structure of a 17 kDa Hh fragment active in the initiation of auto-processing is solved (Figure 1E). The Hh residues Cys-258, His-329, Thr-326, and Asp-303 are involved in auto-processing (Hall et al., 1997). Mutants of these residues were tested for autoprocessing activity by inducing with dithiothreitol (DTT) and/or cholesterol. The DTT was used for the initial steps in the auto-processing reaction of Hh, the thioester formation, whereas cholesterol was used for the next step, the cholesterol transfer. Alanine mutants of Cys-258, His-329, Thr-326 lost the activity in both DTT and cholesterol assays, whereas D303A mutant was only active with DTT but not with cholesterol (Hall et al., 1997).

The compound ST044643 can be used as a positive control as it is active with an IC_50_ of 5 µM in a cholesterolysis assay based on protein fluorescence resonance energy transfer (FRET) (Owen et al., 2015). Nucleophiles such as DTT, glutathione, and hydroxylamine, can stimulate Hh autoprocessing *in vitro*, similar to cholesterol (Hall et al., 1997). The hedgehog autoprocessing is also inhibited by Zn^2+^ with a Ki of 2 μM *in vitro* and at the cellular environment in primary rat astrocyte culture (Xie et al., 2015).

Another area of research that employs the intein, especially the split intein, is gene therapy and gene delivery system. Although AAV-mediated gene delivery is approved, the size of the gene is a limiting factor. The split intein-based techniques could deliver genes above 5 kb in size by a dual vector. The use of split intein to deliver larger genes are gaining importance recently. The immune response due to presence of external intein fragments can be overcome by adding degrons which facilitate the ubiquitin-mediated degradation of the spliced intein.

The *Nanoarchaeum equitans* DNA polymerase (Pol) and *Pyrococcus* sp (GBD strain) Pol inteins are the only 2 reported cases of cysteine-less split inteins. They have inferior splicing properties under native and ambient conditions (Bhagawati et al., 2019). Because of the cysteine residues, the cysteine-containing split intein splicing requires a reducing environment. The reducing agents such as DTT and TCEP may affect the fused protein such as antibodies. The activity of cysteine-dependent inteins may be limited by oxidizing environments like the endoplasmic reticulum, bacterial periplasmic space, or the *in vivo* extracellular milieu. In order to overcome this issue, a cysteine-less split intein was designed and active at ambient temperatures and in the absence of reducing agents, without requiring a denaturation step (Bhagawati et al., 2019; Basturea, 2021). The point of splitting of the target protein to fuse with split intein fragments is also important to get active protein after trans-splicing (Purde et al., 2020). Care must be taken to expose the least hydrophobic residues.

To conclude, although intein was discovered many years ago, the use of it in drug discovery, therapeutics and as a biotechnological tool is of recent origin and is very promising. More efforts are needed to screen and optimize lead intein splicing inhibitors and to develop split inteins in clinical applications.

## Abbreviations

4-HT, 4-hydroxytamoxifen; A549, adenocarcinomic human alveolar basal epithelial cells; AAV, Adeno-associated viral; AAV, Adeno-associated virus vector; ABCA4, ATP Binding Cassette Subfamily A Member 4; ABEs, Adenine base editors; ALS, Amyotrophic lateral sclerosis; CBEs, cytosine base editors; DTA, Diphtheria toxin catalytic domain; DTT, Dithiothreitol; ecDHFR, E. coli dihydrofolate reductase; ER, Estrogen receptor; FRET, Fluorescence resonance energy transfer; GFP, Green fluorescent protein; GLT1, glutamate synthase; HE, homing endonuclease; HER2/ERBB2, Human epidermal growth factor receptor 2; Hh, Hedgehog; KD, Dissociation constant; MDR, multi-drug resistant; MIC, minimum inhibitory concentration; SDS-PAGE, Sodium dodecyl sulphate–polyacrylamide gel electrophoresis; SICLOPPS, Split-intein circular ligation of peptides and proteins; SOD1, Superoxide dismutase 1; SPR, Surface plasmon resonance; TA, Toxin-antitoxin; TB, tuberculosis; TCEP, Tris (2-carboxyethyl) phosphine; ThrRS, Threonyl-tRNA synthetase; VMA, Vacuolar ATPase subunit.
